# Uncertainty quantification of U‐Net based segmentation tool using conformal prediction

**DOI:** 10.1002/acm2.70663

**Published:** 2026-06-23

**Authors:** Bailey J. Borden, John B. Graham‐Knight, Patricia Lasserre, Sarah Lucas, Rasika D. Rajapakshe

**Affiliations:** ^1^ The University of British Columbia ‐ Okanagan Campus Kelowna British Columbia Canada; ^2^ BC Cancer ‐ Kelowna Kelowna British Columbia Canada

**Keywords:** automation, breast, computer vision, CT, deep learning, machine learning, model uncertainty, segmentation

## Abstract

**Background:**

The radiation therapy treatment process is very labour intensive, and artificial intelligence (AI) based auto contouring tools are increasingly being adopted to improve efficiency. However, current acceptance testing of AI auto contouring algorithms relies primarily on area‐ and distance‐based metrics, with limited assessment of model uncertainty.

**Purpose:**

To demonstrate conformal prediction as a complementary error analysis technique for AI auto contouring algorithms, providing spatially localized uncertainty information that traditional metrics do not capture.

**Methods:**

A U‐Net architecture with a ResNet‐34 encoder was trained on BC Cancer breast data to segment the left lung, right lung, and the heart. Initial testing was performed on a subset of 376 computed tomography (CT) scans using both area‐based (IoU) and distance‐based (HD95) metrics. Conformal prediction using adaptive prediction sets was then performed on 138 CT scans. The change in the derivative of the intersection over union (IoU) between the original predictions and the conformal predictions was observed with respect to the selected confidence level.

**Results:**

U‐Net achieved a mean IoU of 0.924 and a mean HD95 of 11.35. When conformal prediction was applied using a 90% confidence threshold, the percent differences between the conformal prediction IoUs and the U‐Net prediction IoUs were 1.01%, 0.89%, and 1.46% for the left lung, right lung, and the heart, respectively. The IoU derivatives differed significantly between true positive and false positive structure predictions (*P* < 0.001), indicating that conformal prediction can distinguish reliable predictions from unreliable ones.

**Conclusions:**

Conformal prediction provides an additional tool for acceptance testing of AI auto contouring algorithms. Beyond traditional area‐ and distance‐based metrics, it spatially localizes uncertain predictions and offers a mechanism for identifying false positives.

## 1 INTRODUCTION

Cancer incidence is increasing in Canada, with an estimated 45% of Canadians expected to be diagnosed within their lifetimes.^1^ Of these, approximately half will receive radiation therapy.^2^ Treatment planning is labor intensive, and automated tools have been introduced over the years to improve efficiency. More recently, artificial intelligence (AI) has entered the workflow in the form of AI‐assisted organ at risk (OAR) contouring,^3^, ^4^, ^5^, ^6^, ^7^, ^8^ allowing additional structures to be contoured automatically compared to what atlas‐based methods could handle. But contours directly influence plan generation and dose calculations.^9^ AI‐generated contours are reviewed by dosimetrists and radiation oncologists before plan generation, but AI decision support tools can still induce automation bias, where clinicians accept incorrect suggestions rather than exercising independent judgment.^10^ Adequate acceptance testing before clinical deployment is therefore essential.

Acceptance testing of AI auto contouring algorithms typically involves both area‐based metrics (e.g., Dice Similarity Coefficient, Intersection over Union (IoU)) and distance‐based metrics (e.g., Hausdorff Distance) evaluated against reference standard contours.^11^, ^12^, ^13^, ^14^ These metrics are well established for assessing contour agreement, but they say nothing about where the model is confident and where it is not.^6^, ^7^, ^8^, ^15^ A contour can achieve a high DSC while containing localized regions of uncertainty at organ boundaries^16^ or where the model predicts a structure absent from the reference standard. Contour deviations near dose gradients affect calculated OAR doses even when overall agreement is high. Error analysis that provides spatially resolved uncertainty information is needed alongside these metrics.

The existing alternatives for uncertainty quantification in deep learning have practical drawbacks. Monte Carlo (MC) dropout approximates Bayesian inference by applying dropout at test time across multiple forward passes and measuring prediction variance.^17^ Deep ensembles train multiple independent models and use disagreement across predictions as an uncertainty estimate.^18^ Both have been applied to medical image segmentation,^19^ but they require either architectural modifications or training and storing multiple models, and neither provides formal statistical guarantees on coverage. Conformal prediction is different.^20^ It is a distribution‐free, post‐hoc framework that operates on saved model predictions, so it can be applied to any auto contouring algorithm without touching the model.^21^ For segmentation, it produces pixel‐level prediction sets with spatially resolved uncertainty, and because it uses saved predictions it can be rerun at multiple confidence levels without rerunning the model.^21^, ^22^ Adaptive prediction sets, a variant for classification tasks, include classes in order of decreasing predicted probability until the cumulative probability exceeds a calibrated threshold.^23^

In this work, we train a U‐Net model on breast radiotherapy CT data to segment the left lung, right lung, and heart, and apply conformal prediction with adaptive prediction sets as an error analysis tool alongside traditional area‐ and distance‐based metrics. The resulting uncertainty maps identify regions where the model is less confident, including structure boundaries and false positive predictions. Through derivative analysis, we show that conformal predictions behave differently for true positive and false positive structures (*P* < 0.001), providing a way to flag unreliable predictions. We discuss how these maps can be integrated into clinical acceptance testing and contour review.

## 2 METHODS

**FIGURE 1:**
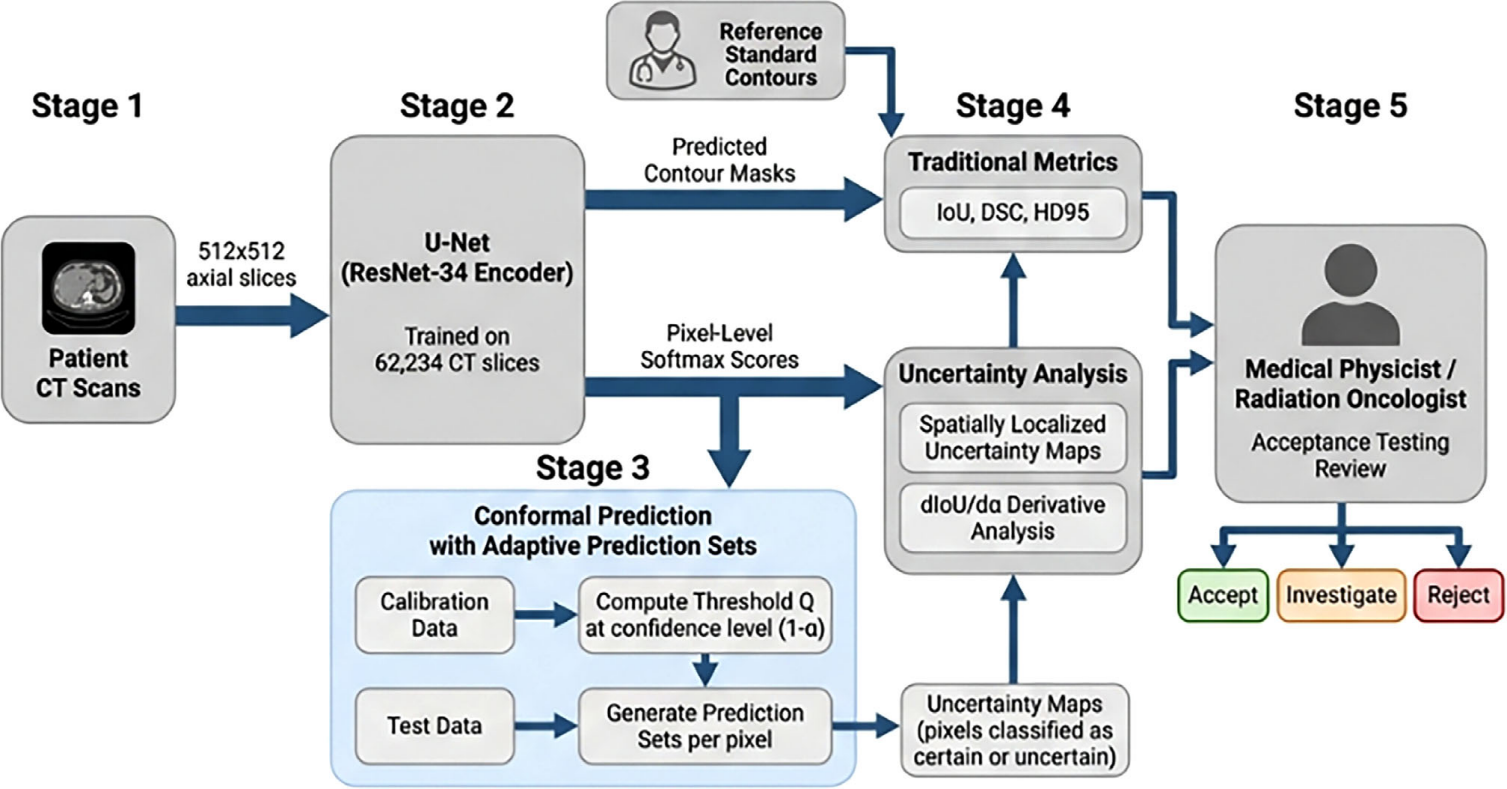
Overview of the methodology used in this work. Patient CT scans are segmented by the U‐Net model, which produces predicted contour masks and pixel‐level softmax scores. The softmax scores are used as input to the conformal prediction pipeline, where a calibration set is used to compute the nonconformity threshold Q at a user‐specified confidence level (1 − *α*), and adaptive prediction sets are generated on the test data to produce spatially localized uncertainty maps. The medical physicist or radiation oncologist reviews both the traditional metrics (IoU, DSC, HD95) and the uncertainty maps as part of the acceptance testing process.

An internally trained U‐Net model was used to demonstrate adequate testing and to discuss error analysis within the predictions of AI models. Conformal prediction using adaptive prediction sets was selected to be used to perform error analysis on the predicted contours.^23^ An overview of the methodology is shown in Figure 1.

The deep learning model selected for this work was a U‐Net architecture with a ResNet‐34 backbone on the encoder path of the network. Pretrained model weights for classification of the ImageNet dataset were used for the ResNet encoder, prior to training to perform organ segmentation.^24^ U‐Net was selected as a well‐established segmentation architecture,^25^ allowing the analysis to focus on the conformal prediction methodology rather than on segmentation performance. The segmentation algorithm was trained on single CT slices of 512 × 512 pixels using data retrospectively collected between 2001 and 2019 from across BC Cancer Centers. Breast radiotherapy data was selected due to the high volume of breast cancer treatments and the resulting large dataset. A total of 62 234 CT slices were used for training the model, 2,063 CT slices were used for validating the model during training, and 13 531 CT slices were used for testing the model. No data augmentation was performed. The CT slices within each data subset all originated from the same CTs, meaning that CT slices from the same patient only existed within a single subset. All data used in the further exploration of the U‐Net model came from the set reserved for testing U‐Net and, as such, had no direct influence on the model's performance. For the purpose of training U‐Net, all non‐breast treatments and any palliative breast cases were excluded from the study cohort and only slices containing reference standard contours were used. To test the use of U‐Net on the selected data set, U‐Net was evaluated on the 13 531 CT slices in the test set. The mean IoU, heart IoU, left lung IoU, and right lung IoU scores on the test set were recorded.

**FIGURE 2:**
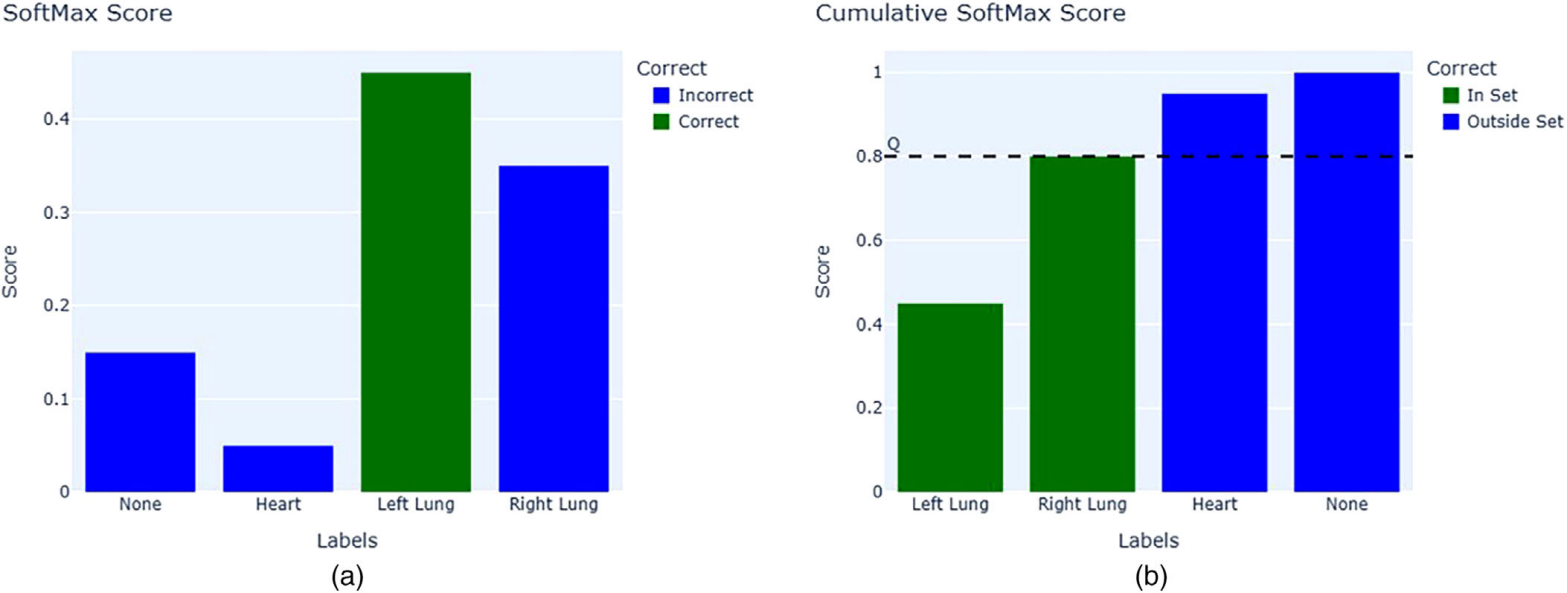
Example of cumulative SoftMax Scores, where SoftMax Scores (a) are ordered from greatest to least and then summed (b) to create Cumulative SoftMax Scores. All classes below the value Q are included in the adaptive prediction set.

**FIGURE 3:**
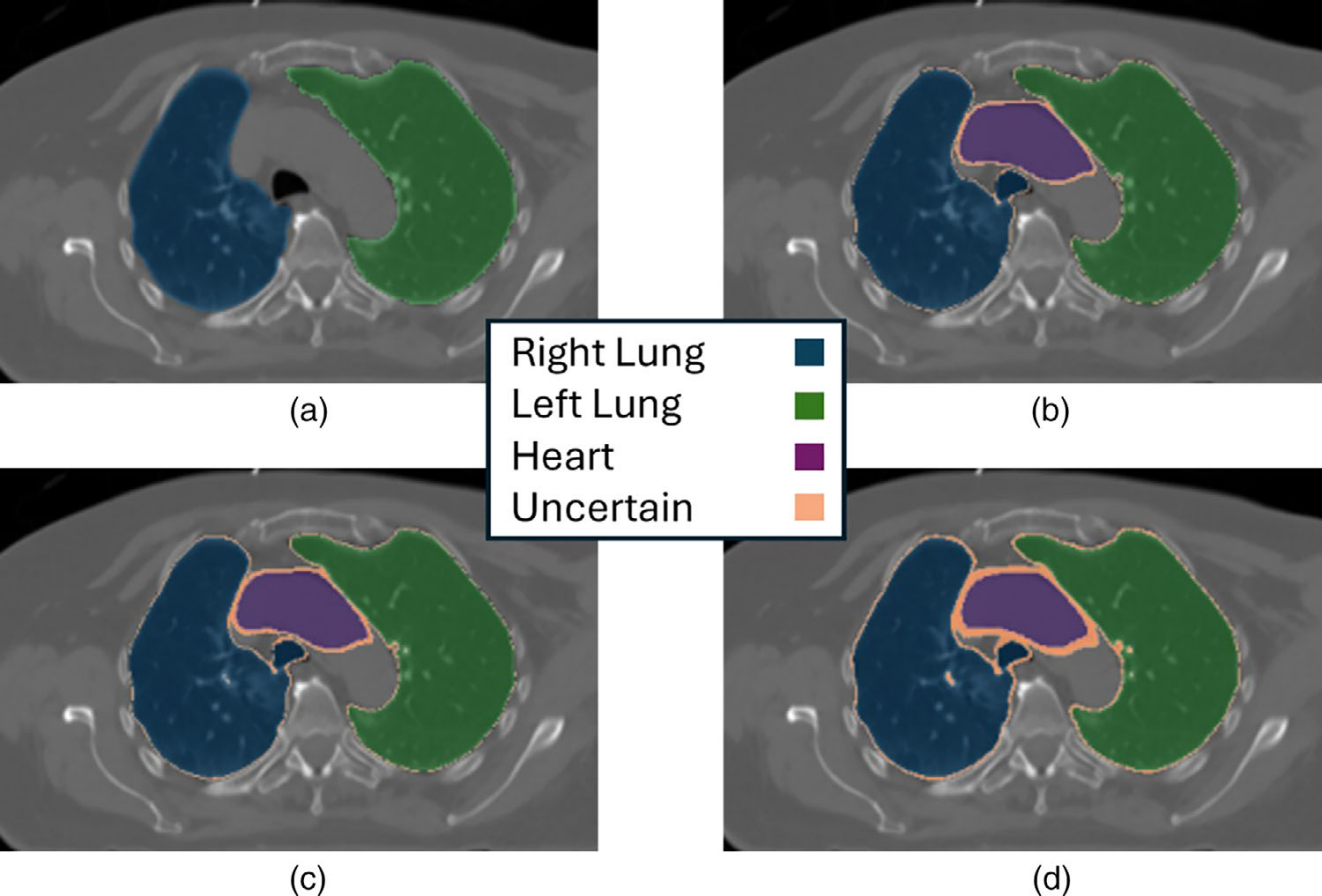
Visualization of reference standard contours (a) and conformal prediction sets overlayed on the original CT image. Solid colors represent predictions within the 90% (b), 95% (c), and 99%(d) coverage requirement, while orange pixels represent uncertain pixels. A legend for each structure color is shown. This example shows uncertain regions within this slice for all three structures. The heart is not contoured in (a), while the U‐Net predictions show a heart mask in the image. It is notable that relative to the size of the predicted heart structure, a larger amount of the structure is uncertain in the heart than in either lung. As the conformal prediction requirements become more strict, the relative area of uncertainty in the heart increases faster than in the lungs.

To perform conformal prediction, 26 497 slices from the test set used to evaluate U‐Net training were selected at random, allowing predictions on slices without reference standard masks. Predictions were made on every axial slice within each CT scan and stored, allowing conformal prediction to be re‐run for various confidence levels. To allow the application of conformal prediction to the predictions, the cumulative softmax pixel output of the true class was selected as the non‐conformity measure. This score was chosen because it directly reflects the model's confidence in its pixel‐level classification and is compatible with the adaptive prediction set framework, where classes are included in the prediction set in order of decreasing softmax probability until a calibrated threshold is reached. Cumulative softmax score is defined as the ordered set of softmax scores, where the set is initially sorted in descending order of probabilities and each element is the sum of all previous elements, such that the largest score is 1. The adaptive prediction set methods create prediction sets including all classes below a threshold value Q which is determined by the distribution of calibration data, and the user defined confidence level. An example of cumulative softmax scores is shown in Figure 2 b. The pixels from 2,407 slices were used to calibrate the conformal predictions, resulting in a final test set of 24 090 slices. An initial *α* value of 0.1 was selected for the primary analysis, corresponding to a 90% coverage requirement. The effects of varying *α* were analyzed across 200 equally spaced values over the interval [0.1, 0.005], and results at three representative levels (*α* = 0.1, 0.05, and 0.01, corresponding to 90%, 95%, and 99% coverage) are shown visually in Figure 3.

To assess whether conformal prediction could distinguish between true positive and false positive structure predictions, the derivative of IoU with respect to *α* was computed for each prediction across the range of *α* values. The maximum value of the normalized derivative $\frac{dIoU}{d\alpha }\cdot \frac{1}{\alpha }$ was recorded for each predicted structure, and the distributions of these maximum values were compared between true positive and false positive predictions using the Mann–Whitney U test. Predictions were classified as true positive if a corresponding reference standard contour existed and as false positive if no reference standard contour was present for that structure on the given slice.

The non‐conformity score was applied to all predictions in the calibration data set. Then the non‐conformity score corresponding to the quantile defined by Equation 1 was determined

(1) $$D=\frac{\left\lceil \left(n+1\right)\left(1-\alpha \right)\right\rceil }{n},$$

where *n* is the total number of pixels present, and *α* is the user selected threshold value between 0 and 1. Once the non‐conformity score associated with the *α* defined quantile was established, prediction sets were made from predictions on the test set using adaptive prediction sets. The predictions in the test set were analyzed in two ways: (1) using the IoU values of the original predictions and the IoU values of the conformal predictions, and (2) visualizing conformal predictions on the original CT images.

For statistical analysis, the IOUs between conformal predictions and the original model predictions were analyzed over 200 equally spaced *α* values over the interval [0.1,0.005]. An initial *α* value of 0.1 was selected for the primary analysis, corresponding to a 90% coverage requirement. This value was chosen as it represents a clinically meaningful confidence level while maintaining sufficient sensitivity to identify uncertain regions. The effects of varying *α* values on the predictions were also analyzed across the full range, allowing the behavior of the conformal predictions to be studied as a function of confidence level. The maximum values of the derivative $\frac{dIoU_{prediction}}{d\alpha }$ were observed, in addition to plotting $\frac{dIoU_{prediction}}{d\alpha }\cdot \frac{1}{\alpha }$ to observe the relative change in IoU per *α*. Observing alpha over this range allows the change in confidence of the model represented by $\frac{dIoU}{d\alpha }$ to be observed.

## 3 RESULTS

The results of U‐Net in the test set are shown in Table 1. U‐Net achieved the lowest IoU in the heart and the highest IoU in the lungs. The heart masks have the lowest HD95 values (Table 1). Overall, the model shows high overlap and reasonable distance to agreement across the three structures. The IoU values in Table 1 are consistent with published benchmarks for U‐Net‐based thoracic OAR segmentation.^26^

**TABLE 1:** Mean performance of U‐Net on 376 CTs.

Model	Mean	Heart	Left lung	Right lung
IoU	0.924	0.877	0.911	0.914
HD95	11.35	8.77	11.37	13.62

Applying conformal prediction at *α* = 0.1 reduced the IoU scores for all three structure classes (Table 2). The heart had the largest reduction, with a 1.46 % difference between the full prediction IoU and the conformal prediction IoU. Figure 3 shows three visualizations of conformal prediction sets, with uncertain pixels in orange. The example is a false positive heart prediction: U‐Net predicts a heart contour where no reference standard exists. Uncertain regions appear along the boundaries of all three structures, but U‐Net is visibly less certain about the heart boundary than either lung. Of the slices selected for conformal predictions, 11,860 contained both lung contours, and 3,409 of these had false positive heart predictions. There were 5,519 slices with heart contours present.

**TABLE 2:** IoU values for the empirical conformal prediction experiment.

Structure	Full prediction IoU	90% Coverage IoU	%Difference
Heart	0.874	0.861	1.46
Left Lung	0.894	0.885	1.01
Right Lung	0.896	0.893	0.894

The 1.01%–1.46% IoU reductions are small in absolute terms, which is expected since conformal prediction only removes the least confident pixels. The heart shows the largest reduction. This makes sense: it is the hardest of the three structures to segment in breast radiotherapy data because it is not always fully visible in the CT scan and contouring practices vary across treatment lateralities.^27^ The lungs, with stronger contrast boundaries, show smaller reductions. What is worth noting is that the ordering of uncertainty (heart, then left lung, then right lung) is not apparent from IoU and HD95 alone. The three confidence levels in Figure 3 (*α* = 0.1, 0.05, 0.01) show more pixels classified as uncertain as *α* decreases.

**FIGURE 4:**
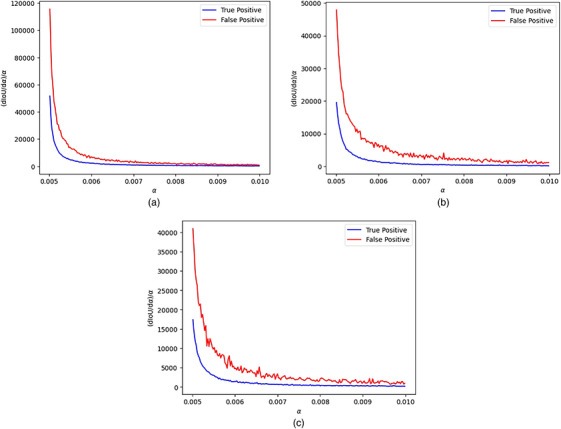
Average relative change in IoU with respect to *α* for false positives and true positives for the heart (a), left lung (b) and right lung (c). The maximum value for the average false positives is over 2.3 times the maximum value for the average true positives in all three cases.

**FIGURE 5:**
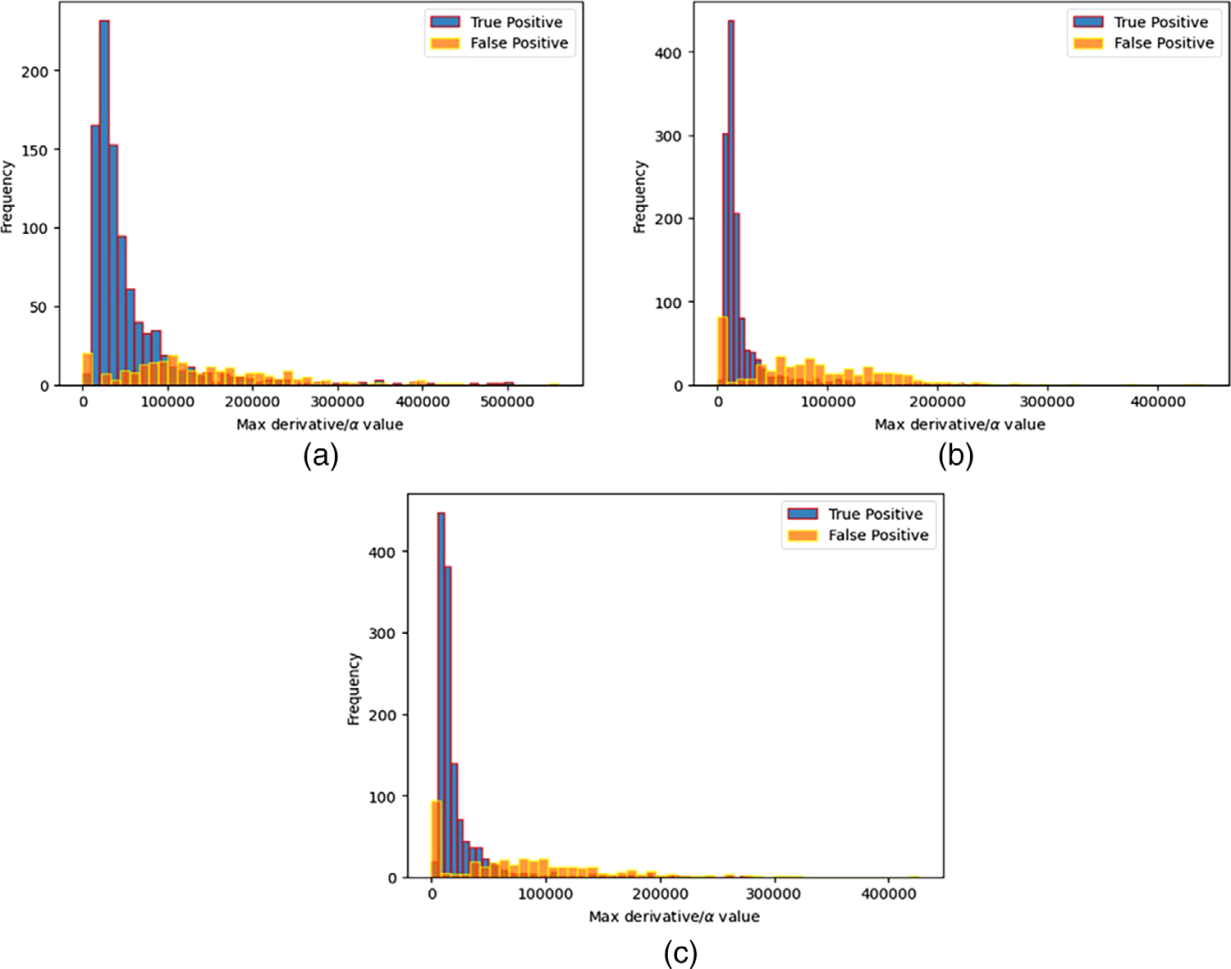
Distribution of maximum derivatives for varying levels of *α* for heart predictions (a), left lung predictions (b), and right lung predictions (c). True positives in this context refer to the predicted structure existing in a given slice, as opposed to a given pixel.

We visually inspected randomly selected axial slices for the change in IoU slope with respect to *α* and noticed that the maximum relative change was consistently larger for false positive predictions than for true positives. Figure 4 confirms this: on average, false positive cases show a significantly higher relative change in slope. The distributions of maximum derivatives (Figure 5) differ between the two groups for all three structures (Mann–Whitney U test, P < 0.001). Table 3 estimates the effect of thresholding at the 90th percentile of the true positive distributions. For the heart, this reduces 136 false positives while introducing 94 false negatives, a net reduction of 42 errors. The lungs show a better trade‐off, with net reductions of 194 (left) and 120 (right). In all three structures the decrease in false positives exceeds the increase in false negatives, though the optimal threshold would need to be set per structure.

**TABLE 3:** Potential reductions in false positives based on a thresholding using the 90th percentile value of the distribution of maximum true positive derivatives.

Structure	Heart	Left lung	Right lung
Total true positives	944	1296	1290
Total false positives	237	448	373
Total errors reduced	42	194	120
Increase in false negatives	94	130	129
Decrease in false positives	136	324	249

## 4 DISCUSSION

Of the three structures, the heart shows the most uncertainty: the IoU drops 1.46% between conformal and original predictions, compared to 1.01% and 0.894% for the left and right lungs. The conformal maps show that uncertain regions cluster along structure boundaries, consistent with the well‐documented difficulty of organ boundary delineation.^16^ This is where the clinical relevance becomes clear. In radiation therapy, organ boundaries can sit near steep dose gradients, and even small contour shifts there can meaningfully change the calculated dose to the target and OARs.^27^, ^28^ A contour error in a high‐dose gradient region carries more risk than one in a flat‐dose region, even if both contribute equally to aggregate IoU. Conformal prediction identifies exactly these boundary regions.

The U‐Net model frequently predicts the heart where no reference standard contour exists. Of the 11,860 slices with both lung contours present, 3,409 (28.7%) contained false positive heart predictions. In breast radiotherapy, OAR contouring extent varies with treatment laterality, technique, and clinical risk assessment,^27^, ^28^ and practices changed over the collection period. The model cannot distinguish these sources of variation; it reproduces whatever contours appear in the training data. A 28.7% false positive rate would be hard to catch during routine contour review without systematic analysis. While such a poorly performing model should be prevented from clinical adoption, a rate this high should also be flagged during acceptance testing and prompt investigation into the training data composition. Conformal prediction flags these regions with elevated uncertainty, giving reviewers a starting point.

False predictions on the lungs look different. Visual inspection showed that the model contoured some air cavities outside the lungs (trachea, bronchi, bowel loops) as lung tissue. This is not surprising: these structures have similar Hounsfield unit profiles to lung parenchyma, and the model lacks spatial reasoning to tell them apart.^29^ The conformal prediction maps flagged elevated uncertainty in these regions, with noticeably lower softmax confidence than predictions within the true lung boundaries.

As shown in Figure 3, U‐Net predicts structures even when no reference standard contour exists. At *α* = 0.05, a large portion of this false structure is already marked as uncertain, and at *α* = 0.01 it grows further. The false positive pixels get progressively stripped away as *α* decreases.

The derivative analysis provides the most actionable result. As shown in Figure 4, the relative change in $\frac{dIoU}{d\alpha }$ is consistently higher for false positives than for true positives, and the Mann–Whitney U test confirms this (*P* < 0.001, Figure 5). The true positive distributions are much tighter than the false positive distributions for all three structures. Table 3 shows what happens in practice: thresholding at the 90th percentile of the true positive distributions reduces false positives across all three structures, though at the cost of some new false negatives. In all cases the trade‐off is favorable. These thresholds need validation on independent data.

The tight true positive distributions tell us something on their own: the model behaves consistently when it gets things right. Wrong predictions, by contrast, produce wider, higher‐magnitude derivatives. A prediction whose derivative falls well outside the true positive distribution can be flagged for review without relying on visual inspection alone. Where exactly to set the threshold depends on the structure and clinical context, and prospective validation will be needed.

From a workflow perspective, conformal prediction does not add new numbers for clinicians to learn. It adds a visual overlay. During acceptance testing, uncertainty maps for a representative case set can be reviewed alongside contours to spot systematic areas of low confidence. During routine QA, an automated overlay can flag regions for the reviewing dosimetrist or radiation oncologist. A standardized test suite across patient anatomies and treatment lateralities would let the medical physicist compare uncertainty patterns across cases and catch systematic model weaknesses that case‐by‐case review would miss.

The practical advantage of conformal prediction over other uncertainty methods is clearest in deployment. MC dropout requires 10–50 forward passes at test time,^17^ which is substantial when processing hundreds of CT slices per patient. Deep ensembles need multiple independently trained models.^18^ Both derive uncertainty from the model's own representations, so if the model is poorly calibrated, the estimates will be too. Conformal prediction uses a held‐out calibration set instead, and its coverage guarantee holds regardless of calibration.^21^ For acceptance testing, statistical guarantees matter more than heuristic estimates. This matters even more for commercial auto contouring systems,^30^ where medical physicists have no access to model architectures, weights, or intermediate representations. MC dropout and Bayesian methods both require model internals. Conformal prediction needs only the outputs and a calibration dataset. That said, the two approaches could be combined: MC dropout variance or ensemble disagreement could serve as nonconformity scores within the conformal framework, preserving the coverage guarantee while using richer uncertainty signals.

The biggest practical barrier is the reliance on softmax outputs. Commercial auto contouring products may not expose softmax scores, which means a different nonconformity score would be needed. Morphological prediction sets applied to binary segmentation outputs are one option,^31^ and adapting the methodology for commercial systems is where we plan to focus next.

It is possible that the model is picking up out‐of‐distribution pixels which has possible implications for smaller cancers such as nasopharyngeal carcinoma. While this was not analyzed in this work, it is a topic of interest in future work.

The dataset also lacked patient demographic information, preventing subgroup analysis. The data was collected from multiple centers across the province of British Columbia, but without demographics we cannot assess whether model performance or uncertainty patterns differ across patient populations. Such testing should precede clinical deployment.^32^, ^33^, ^34^

The U‐Net model analyzed here segments only three structures, with the left and right lungs separated to provide clinical utility. The limited structure set was dictated by available data. Extending to more complex structures such as the axilla lymph nodes, and testing whether the true/false positive distinction holds for larger structure sets, is an obvious next step.

The training data spans 2001–2019, a period over which contouring practices changed substantially.^35^ Treatment techniques, imaging protocols, and guidelines all evolved, so the reference standard contours used for training are heterogeneous. The model likely learned a mixture of historical practices rather than any single current guideline, which probably contributes to the false positive heart predictions we observed. The conformal prediction calibration step absorbs some of this variability because the calibration and test sets share the same distributional characteristics, but evaluating on datasets with more uniform contouring standards would help isolate the effect.

## 5 CONCLUSION

Current acceptance testing of AI auto contouring algorithms relies on area‐ and distance‐based metrics that measure overall contour agreement but say nothing about where the model is uncertain. We showed that conformal prediction with adaptive prediction sets fills this gap. The uncertainty maps localize low‐confidence regions to structure boundaries and false positive predictions, and the derivative analysis can distinguish between the two (*P* < 0.001). Thresholding on derivative distributions reduced false positives across all three structures (Table 3).

Several limitations apply. We used a relatively simple architecture (U‐Net with ResNet‐34); more advanced models like nnU‐Net^36^ will show different uncertainty characteristics. The methodology has not been validated in clinical practice. The reliance on softmax outputs prevents direct application to commercial systems, though morphological prediction sets offer a path forward.^31^ And the analysis covers one anatomical site with three structures.

The broader motivation is automation bias. Dratsch et al. showed that AI decision support reduces diagnostic accuracy even among experienced radiologists,^10^ and Graham‐Knight et al. found that radiologists and AI have complementary strengths in mammographic cancer detection.^37^ Presenting AI predictions without uncertainty information encourages passive acceptance. Conformal prediction maps give clinicians something concrete to scrutinize.

Future works include testing on more advanced architectures, state‐of‐the‐art models, additional anatomical sites, and prospective clinical validation, and adapting the approach for commercial systems using output‐based nonconformity scores.

## AUTHOR CONTRIBUTIONS

**Bailey J. Borden**: Conceptualization; methodology; software; formal analysis; writing—original draft. **John B. Graham‐Knight**: Methodology; software; validation; writing—review & editing. **Patricia Lasserre**: Supervision; writing—review & editing. **Sarah Lucas**: Validation; resources. **Rasika D. Rajapakshe**: Conceptualization; supervision; funding acquisition; writing—review & editing.

## CONFLICT OF INTEREST STATEMENT

The authors declare no conflicts of interest.

## ETHICS STATEMENT

This work was approved by BC Cancer‐UBC research ethics (approval H18‐01183).

## GENERATIVE AI STATEMENT

The use of generative artificial intelligence within this work was limited to aiding with corrections to grammar.

## Data Availability

Due to provincial privacy and ethical requirements, the data used in this research cannot be made public. Requests to access this data must be made through the BC Cancer‐UBC research ethics board approval process.
